# What lies underneath: The genetics and neurobiology of psychopathy

**DOI:** 10.1192/j.eurpsy.2021.1179

**Published:** 2021-08-13

**Authors:** M. Lemos, T. Queirós

**Affiliations:** Psychiatry, Centro Hospitalar Lisboa Norte, Lisboa, Portugal

**Keywords:** anti-social personality disorder, psychopathy, Neurobiology, Pathophysiology

## Abstract

**Introduction:**

Psychopathy is a personality disorder characterized by lack of empathy, grandiosity, an impulsive lifestyle and antisociality. Anti-social personality disorder (ASPD) and psychopathy are distinct concepts presenting different criteria. Most people with a diagnosis of psychopathy also meet criteria for ASPD while the reverse is not true. Along the years there has been an increasing interest in investigating genetic and neurobiological factors.

**Objectives:**

To analyze the neurobiological factors involved in psychopathy and anti-social personality disorder according to the scientific knowledge available.

**Methods:**

Review of scientific literature via PubMed search, using the terms “anti-social personality disorder”, “biology or etiology or pathophysiology and psychopathy”.

**Results:**

The strongest evidence base for a genetic pathway is associated with the low-expression variant of the Monoamine Oxidase-A (MAO-A) which is linked to the X chromosome. Other genetic factors involve the 5-HTT gene, dopamine receptor genes (DRD4 and DRD2) and genetic polimorfisms at SNAP25 t-snare protein, OXT gene and the CNR1 and FAAH cannabinoid receptor gene. Structural differences in the brain have been noticed such as reduced gray matter volume in the orbitofrontal cortex, gray matter volume reductions in the mid-anterior insula and left anterior temporal cortex, subtle reductions in gray matter volume across several paralimbic and limbic areas.

**Conclusions:**

There is considerable evidence regarding various possible underlying neurobiological processes in psychopathy although it is insufficient to suggest a single biological etiology and environmental influences cannot be excluded from a complete understanding of this disorder. The neurobiological correlates found hold promise for new research and treatment.

